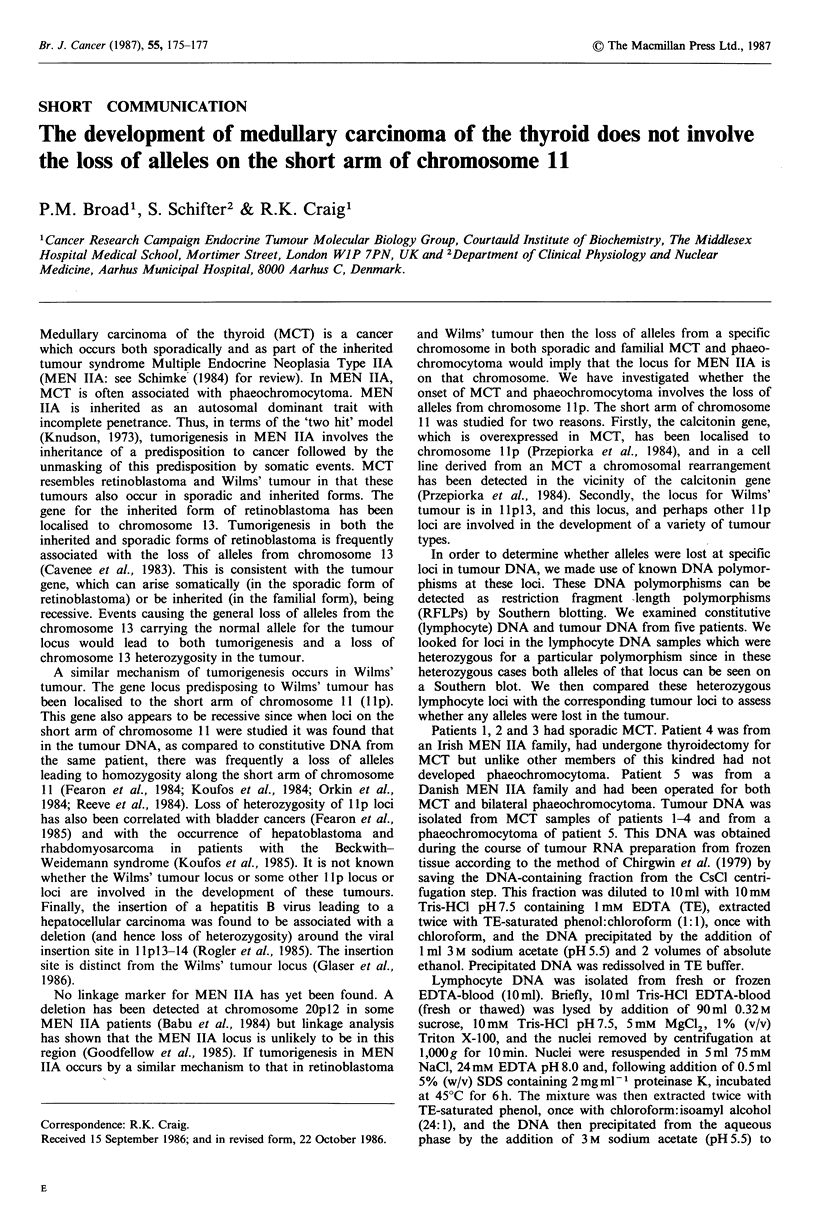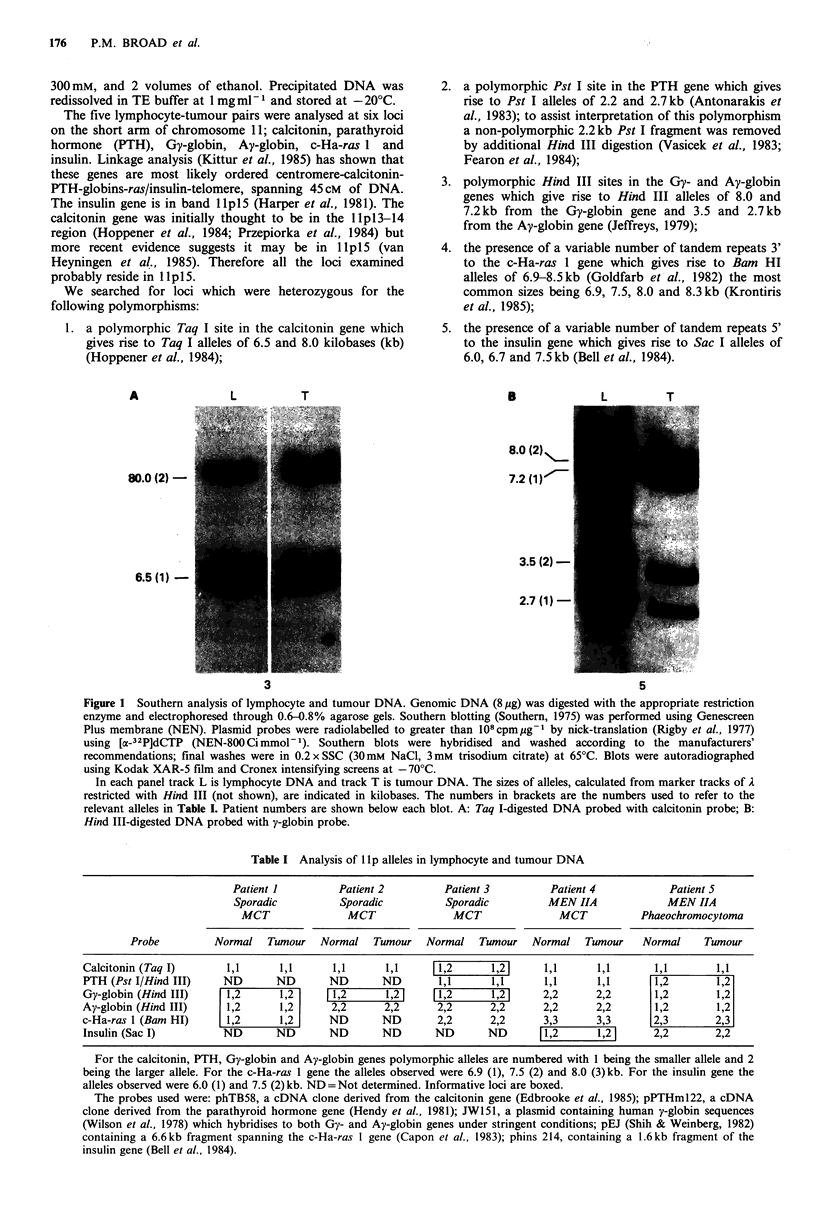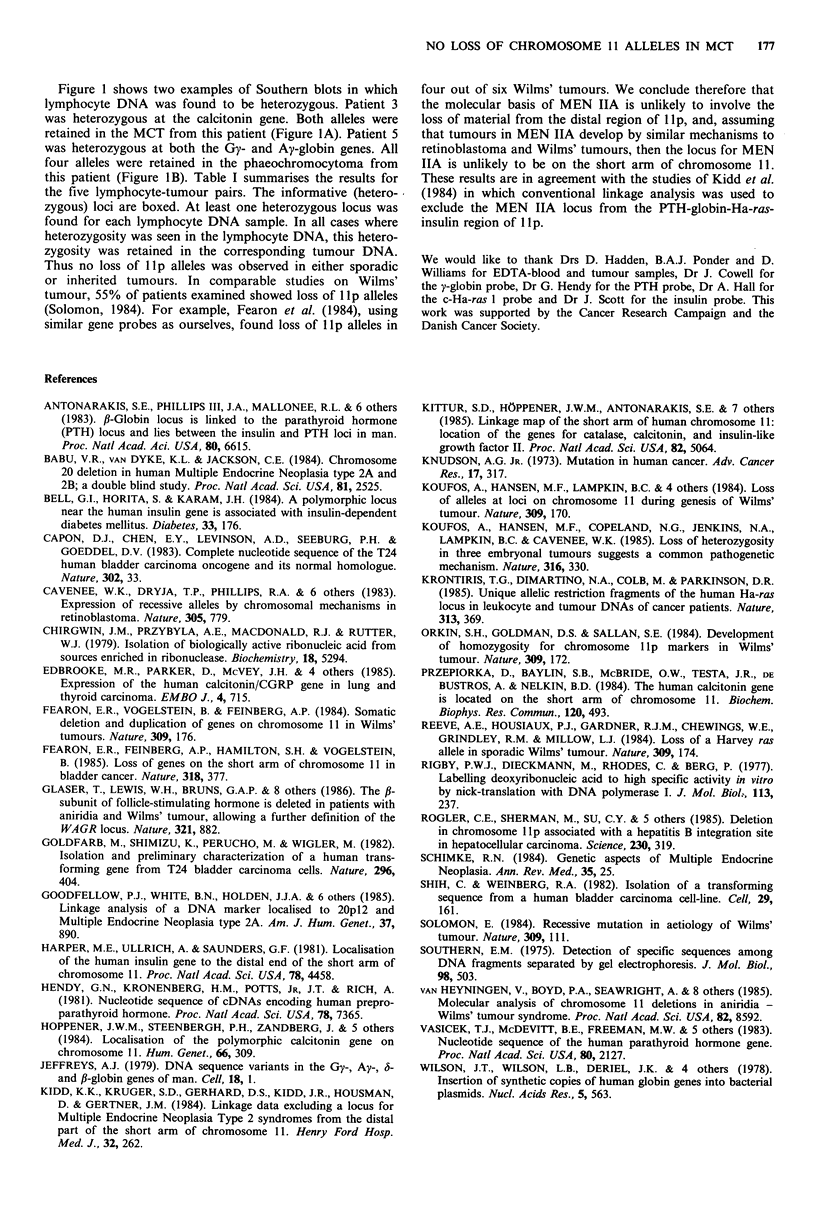# The development of medullary carcinoma of the thyroid does not involve the loss of alleles on the short arm of chromosome 11.

**DOI:** 10.1038/bjc.1987.34

**Published:** 1987-02

**Authors:** P. M. Broad, S. Schifter, R. K. Craig

## Abstract

**Images:**


Br. J. Cancer (1987), 55, 175–177 © The Macmillan Press Ltd., 1987

SHORT COMMUNICATION

### The development of medullary carcinoma of the thyroid does not involve the loss of alleles on the short arm of chromosome 11

P.M. Broad[1], S. Schifter[2] & R.K. Craig[1]

[1]*Cancer Research Campaign Endocrine Tumour Molecular Biology Group, Courtauld Institute of Biochemistry, The Middlesex Hospital Medical School, Mortimer Street, London W1P 7PN, UK and* [2]*Department of Clinical Physiology and Nuclear Medicine, Aarhus Municipal Hospital, 8000 Aarhus C, Denmark.*

Medullary carcinoma of the thyroid (MCT) is a cancer which occurs both sporadically and as part of the inherited tumour syndrome Multiple Endocrine Neoplasia Type IIA (MEN IIA: see Schimke (1984) for review). In MEN IIA, MCT is often associated with phaeochromocytoma. MEN IIA is inherited as an autosomal dominant trait with incomplete penetrance. Thus, in terms of the 'two hit' model (Knudson, 1973), tumorigenesis in MEN IIA involves the inheritance of a predisposition to cancer followed by the unmasking of this predisposition by somatic events. MCT resembles retinoblastoma and Wilms' tumour in that these tumours also occur in sporadic and inherited forms. The gene for the inherited form of retinoblastoma has been localised to chromosome 13. Tumorigenesis in both the inherited and sporadic forms of retinoblastoma is frequently associated with the loss of alleles from chromosome 13 (Cavenee *et al.*, 1983). This is consistent with the tumour gene, which can arise somatically (in the sporadic form of retinoblastoma) or be inherited (in the familial form), being recessive. Events causing the general loss of alleles from the chromosome 13 carrying the normal allele for the tumour locus would lead to both tumorigenesis and a loss of chromosome 13 heterozygosity in the tumour.

A similar mechanism of tumorigenesis occurs in Wilms' tumour. The gene locus predisposing to Wilms' tumour has been localised to the short arm of chromosome 11 (11p). This gene also appears to be recessive since when loci on the short arm of chromosome 11 were studied it was found that in the tumour DNA, as compared to constitutive DNA from the same patient, there was frequently a loss of alleles leading to homozygosity along the short arm of chromosome 11 (Fearon *et al.*, 1984; Koufos *et al.*, 1984; Orkin *et al.*, 1984; Reeve *et al.*, 1984). Loss of heterozygosity of 11p loci has also been correlated with bladder cancers (Fearon *et al.*, 1985) and with the occurrence of hepatoblastoma and rhabdomyosarcoma in patients with the Beckwith–Weidemann syndrome (Koufos *et al.*, 1985). It is not known whether the Wilms' tumour locus or some other 11p locus or loci are involved in the development of these tumours. Finally, the insertion of a hepatitis B virus leading to a hepatocellular carcinoma was found to be associated with a deletion (and hence loss of heterozygosity) around the viral insertion site in 11p13–14 (Rogler *et al.*, 1985). The insertion site is distinct from the Wilms' tumour locus (Glaser *et al.*, 1986).

No linkage marker for MEN IIA has yet been found. A deletion has been detected at chromosome 20p12 in some MEN IIA patients (Babu *et al.*, 1984) but linkage analysis has shown that the MEN IIA locus is unlikely to be in this region (Goodfellow *et al.*, 1985). If tumorigenesis in MEN IIA occurs by a similar mechanism to that in retinoblastoma and Wilms' tumour then the loss of alleles from a specific chromosome in both sporadic and familial MCT and phaeochromocytoma would imply that the locus for MEN IIA is on that chromosome. We have investigated whether the onset of MCT and phaeochromocytoma involves the loss of alleles from chromosome 11p. The short arm of chromosome 11 was studied for two reasons. Firstly, the calcitonin gene, which is overexpressed in MCT, has been localised to chromosome 11p (Przepiorka *et al.*, 1984), and in a cell line derived from an MCT a chromosomal rearrangement has been detected in the vicinity of the calcitonin gene (Przepiorka *et al.*, 1984). Secondly, the locus for Wilms' tumour is in 11p13, and this locus, and perhaps other 11p loci are involved in the development of a variety of tumour types.

In order to determine whether alleles were lost at specific loci in tumour DNA, we made use of known DNA polymorphisms at these loci. These DNA polymorphisms can be detected as restriction fragment length polymorphisms (RFLPs) by Southern blotting. We examined constitutive (lymphocyte) DNA and tumour DNA from five patients. We looked for loci in the lymphocyte DNA samples which were heterozygous for a particular polymorphism since in these heterozygous cases both alleles of that locus can be seen on a Southern blot. We then compared these heterozygous lymphocyte loci with the corresponding tumour loci to assess whether any alleles were lost in the tumour.

Patients 1, 2 and 3 had sporadic MCT. Patient 4 was from an Irish MEN IIA family, had undergone thyroidectomy for MCT but unlike other members of this kindred had not developed phaeochromocytoma. Patient 5 was from a Danish MEN IIA family and had been operated for both MCT and bilateral phaeochromocytoma. Tumour DNA was isolated from MCT samples of patients 1–4 and from a phaeochromocytoma of patient 5. This DNA was obtained during the course of tumour RNA preparation from frozen tissue according to the method of Chirgwin *et al.* (1979) by saving the DNA-containing fraction from the CsCl centrifugation step. This fraction was diluted to 10 ml with 10 mM Tris-HCl pH 7.5 containing 1 mM EDTA (TE), extracted twice with TE-saturated phenol:chloroform (1:1), once with chloroform, and the DNA precipitated by the addition of 1 ml 3 M sodium acetate (pH 5.5) and 2 volumes of absolute ethanol. Precipitated DNA was redissolved in TE buffer.

Lymphocyte DNA was isolated from fresh or frozen EDTA-blood (10 ml). Briefly, 10 ml Tris-HCl EDTA-blood (fresh or thawed) was lysed by addition of 90 ml 0.32 M sucrose, 10 mM Tris-HCl pH 7.5, 5 mM $MgCl_2$, 1% (v/v) Triton X-100, and the nuclei removed by centrifugation at 1,000 *g* for 10 min. Nuclei were resuspended in 5 ml 75 mM NaCl, 24 mM EDTA pH 8.0 and, following addition of 0.5 ml 5% (w/v) SDS containing 2 mg $ml^{-1}$ proteinase K, incubated at 45°C for 6 h. The mixture was then extracted twice with TE-saturated phenol, once with chloroform:isoamyl alcohol (24:1), and the DNA then precipitated from the aqueous phase by the addition of 3 M sodium acetate (pH 5.5) to

Correspondence: R.K. Craig.
Received 15 September 1986; and in revised form, 22 October 1986.

300 mM, and 2 volumes of ethanol. Precipitated DNA was redissolved in TE buffer at 1 mg ml$^{-1}$ and stored at $-20$°C.

The five lymphocyte-tumour pairs were analysed at six loci on the short arm of chromosome 11; calcitonin, parathyroid hormone (PTH), Gγ-globin, Aγ-globin, c-Ha-*ras* 1 and insulin. Linkage analysis (Kittur *et al.*, 1985) has shown that these genes are most likely ordered centromere-calcitonin-PTH-globins-*ras*/insulin-telomere, spanning 45 cM of DNA. The insulin gene is in band 11p15 (Harper *et al.*, 1981). The calcitonin gene was initially thought to be in the 11p13–14 region (Hoppener *et al.*, 1984; Przepiorka *et al.*, 1984) but more recent evidence suggests it may be in 11p15 (van Heyningen *et al.*, 1985). Therefore all the loci examined probably reside in 11p15.

We searched for loci which were heterozygous for the following polymorphisms:

1. a polymorphic *Taq* I site in the calcitonin gene which gives rise to *Taq* I alleles of 6.5 and 8.0 kilobases (kb) (Hoppener *et al.*, 1984);
2. a polymorphic *Pst* I site in the PTH gene which gives rise to *Pst* I alleles of 2.2 and 2.7 kb (Antonarakis *et al.*, 1983); to assist interpretation of this polymorphism a non-polymorphic 2.2 kb *Pst* I fragment was removed by additional *Hin*d III digestion (Vasicek *et al.*, 1983; Fearon *et al.*, 1984);
3. polymorphic *Hin*d III sites in the Gγ- and Aγ-globin genes which give rise to *Hin*d III alleles of 8.0 and 7.2 kb from the Gγ-globin gene and 3.5 and 2.7 kb from the Aγ-globin gene (Jeffreys, 1979);
4. the presence of a variable number of tandem repeats 3' to the c-Ha-*ras* 1 gene which gives rise to *Bam* HI alleles of 6.9–8.5 kb (Goldfarb *et al.*, 1982) the most common sizes being 6.9, 7.5, 8.0 and 8.3 kb (Krontiris *et al.*, 1985);
5. the presence of a variable number of tandem repeats 5' to the insulin gene which gives rise to *Sac* I alleles of 6.0, 6.7 and 7.5 kb (Bell *et al.*, 1984).

**Figure 1** Southern analysis of lymphocyte and tumour DNA. Genomic DNA (8 μg) was digested with the appropriate restriction enzyme and electrophoresed through 0.6–0.8% agarose gels. Southern blotting (Southern, 1975) was performed using Genescreen Plus membrane (NEN). Plasmid probes were radiolabelled to greater than $10^8$ cpm μg$^{-1}$ by nick-translation (Rigby *et al.*, 1977) using [α-$^{32}$P]dCTP (NEN-800 Ci mmol$^{-1}$). Southern blots were hybridised and washed according to the manufacturers' recommendations; final washes were in 0.2 × SSC (30 mM NaCl, 3 mM trisodium citrate) at 65°C. Blots were autoradiographed using Kodak XAR-5 film and Cronex intensifying screens at $-70$°C.

In each panel track L is lymphocyte DNA and track T is tumour DNA. The sizes of alleles, calculated from marker tracks of λ restricted with *Hin*d III (not shown), are indicated in kilobases. The numbers in brackets are the numbers used to refer to the relevant alleles in **Table I.** Patient numbers are shown below each blot. A: *Taq* I-digested DNA probed with calcitonin probe; B: *Hin*d III-digested DNA probed with γ-globin probe.

**Table I** Analysis of 11p alleles in lymphocyte and tumour DNA

| | *Patient 1 Sporadic MCT* | | *Patient 2 Sporadic MCT* | | *Patient 3 Sporadic MCT* | | *Patient 4 MEN IIA MCT* | | *Patient 5 MEN IIA Phaeochromocytoma* | |
|---|---|---|---|---|---|---|---|---|---|---|
| *Probe* | *Normal* | *Tumour* | *Normal* | *Tumour* | *Normal* | *Tumour* | *Normal* | *Tumour* | *Normal* | *Tumour* |
| Calcitonin (*Taq* I) | 1,1 | 1,1 | 1,1 | 1,1 | 1,2 | 1,2 | 1,1 | 1,1 | 1,1 | 1,1 |
| PTH (*Pst* I/*Hin*d III) | ND | ND | ND | ND | 1,1 | 1,1 | 1,1 | 1,1 | 1,2 | 1,2 |
| Gγ-globin (*Hin*d III) | 1,2 | 1,2 | 1,2 | 1,2 | 1,2 | 1,2 | 2,2 | 2,2 | 1,2 | 1,2 |
| Aγ-globin (*Hin*d III) | 1,2 | 1,2 | 2,2 | 2,2 | 2,2 | 2,2 | 2,2 | 2,2 | 1,2 | 1,2 |
| c-Ha-*ras* 1 (*Bam* HI) | 1,2 | 1,2 | ND | ND | 2,2 | 2,2 | 3,3 | 3,3 | 2,3 | 2,3 |
| Insulin (Sac I) | ND | ND | ND | ND | ND | ND | 1,2 | 1,2 | 2,2 | 2,2 |

For the calcitonin, PTH, Gγ-globin and Aγ-globin genes polymorphic alleles are numbered with 1 being the smaller allele and 2 being the larger allele. For the c-Ha-*ras* 1 gene the alleles observed were 6.9 (1), 7.5 (2) and 8.0 (3) kb. For the insulin gene the alleles observed were 6.0 (1) and 7.5 (2) kb. ND = Not determined. Informative loci are boxed.

The probes used were: phTB58, a cDNA clone derived from the calcitonin gene (Edbrooke *et al.*, 1985); pPTHm122, a cDNA clone derived from the parathyroid hormone gene (Hendy *et al.*, 1981); JW151, a plasmid containing human γ-globin sequences (Wilson *et al.*, 1978) which hybridises to both Gγ- and Aγ-globin genes under stringent conditions; pEJ (Shih & Weinberg, 1982) containing a 6.6 kb fragment spanning the c-Ha-*ras* 1 gene (Capon *et al.*, 1983); phins 214, containing a 1.6 kb fragment of the insulin gene (Bell *et al.*, 1984).

Figure 1 shows two examples of Southern blots in which lymphocyte DNA was found to be heterozygous. Patient 3 was heterozygous at the calcitonin gene. Both alleles were retained in the MCT from this patient (Figure 1A). Patient 5 was heterozygous at both the Gγ- and Aγ-globin genes. All four alleles were retained in the phaeochromocytoma from this patient (Figure 1B). Table I summarises the results for the five lymphocyte-tumour pairs. The informative (heterozygous) loci are boxed. At least one heterozygous locus was found for each lymphocyte DNA sample. In all cases where heterozygosity was seen in the lymphocyte DNA, this heterozygosity was retained in the corresponding tumour DNA. Thus no loss of 11p alleles was observed in either sporadic or inherited tumours. In comparable studies on Wilms' tumour, 55% of patients examined showed loss of 11p alleles (Solomon, 1984). For example, Fearon *et al.* (1984), using similar gene probes as ourselves, found loss of 11p alleles in four out of six Wilms' tumours. We conclude therefore that the molecular basis of MEN IIA is unlikely to involve the loss of material from the distal region of 11p, and, assuming that tumours in MEN IIA develop by similar mechanisms to retinoblastoma and Wilms' tumours, then the locus for MEN IIA is unlikely to be on the short arm of chromosome 11. These results are in agreement with the studies of Kidd *et al.* (1984) in which conventional linkage analysis was used to exclude the MEN IIA locus from the PTH-globin-Ha-*ras*-insulin region of 11p.

We would like to thank Drs D. Hadden, B.A.J. Ponder and D. Williams for EDTA-blood and tumour samples, Dr J. Cowell for the γ-globin probe, Dr G. Hendy for the PTH probe, Dr A. Hall for the c-Ha-*ras* 1 probe and Dr J. Scott for the insulin probe. This work was supported by the Cancer Research Campaign and the Danish Cancer Society.

### References

ANTONARAKIS, S.E., PHILLIPS III, J.A., MALLONEE, R.L. & 6 others (1983). β-Globin locus is linked to the parathyroid hormone (PTH) locus and lies between the insulin and PTH loci in man. *Proc. Natl Acad. Aci. USA*, **80**, 6615.

BABU, V.R., VAN DYKE, K.L. & JACKSON, C.E. (1984). Chromosome 20 deletion in human Multiple Endocrine Neoplasia type 2A and 2B; a double blind study. *Proc. Natl Acad. Sci. USA*, **81**, 2525.

BELL, G.I., HORITA, S. & KARAM, J.H. (1984). A polymorphic locus near the human insulin gene is associated with insulin-dependent diabetes mellitus. *Diabetes*, **33**, 176.

CAPON, D.J., CHEN, E.Y., LEVINSON, A.D., SEEBURG, P.H. & GOEDDEL, D.V. (1983). Complete nucleotide sequence of the T24 human bladder carcinoma oncogene and its normal homologue. *Nature*, **302**, 33.

CAVENEE, W.K., DRYJA, T.P., PHILLIPS, R.A. & 6 others (1983). Expression of recessive alleles by chromosomal mechanisms in retinoblastoma. *Nature*, **305**, 779.

CHIRGWIN, J.M., PRZYBYLA, A.E., MACDONALD, R.J. & RUTTER, W.J. (1979). Isolation of biologically active ribonucleic acid from sources enriched in ribonuclease. *Biochemistry*, **18**, 5294.

EDBROOKE, M.R., PARKER, D., McVEY, J.H. & 4 others (1985). Expression of the human calcitonin/CGRP gene in lung and thyroid carcinoma. *EMBO J.*, **4**, 715.

FEARON, E.R., VOGELSTEIN, B. & FEINBERG, A.P. (1984). Somatic deletion and duplication of genes on chromosome 11 in Wilms' tumours. *Nature*, **309**, 176.

FEARON, E.R., FEINBERG, A.P., HAMILTON, S.H. & VOGELSTEIN, B. (1985). Loss of genes on the short arm of chromosome 11 in bladder cancer. *Nature*, **318**, 377.

GLASER, T., LEWIS, W.H., BRUNS, G.A.P. & 8 others (1986). The β-subunit of follicle-stimulating hormone is deleted in patients with aniridia and Wilms' tumour, allowing a further definition of the *WAGR* locus. *Nature*, **321**, 882.

GOLDFARB, M., SHIMIZU, K., PERUCHO, M. & WIGLER, M. (1982). Isolation and preliminary characterization of a human transforming gene from T24 bladder carcinoma cells. *Nature*, **296**, 404.

GOODFELLOW, P.J., WHITE, B.N., HOLDEN, J.J.A. & 6 others (1985). Linkage analysis of a DNA marker localised to 20p12 and Multiple Endocrine Neoplasia type 2A. *Am. J. Hum. Genet.*, **37**, 890.

HARPER, M.E., ULLRICH, A. & SAUNDERS, G.F. (1981). Localisation of the human insulin gene to the distal end of the short arm of chromosome 11. *Proc. Natl Acad. Sci. USA*, **78**, 4458.

HENDY, G.N., KRONENBERG, H.M., POTTS, JR, J.T. & RICH, A. (1981). Nucleotide sequence of cDNAs encoding human preproparathyroid hormone. *Proc. Natl Acad. Sci. USA*, **78**, 7365.

HOPPENER, J.W.M., STEENBERGH, P.H., ZANDBERG, J. & 5 others (1984). Localisation of the polymorphic calcitonin gene on chromosome 11. *Hum. Genet.*, **66**, 309.

JEFFREYS, A.J. (1979). DNA sequence variants in the Gγ-, Aγ-, δ- and β-globin genes of man. *Cell*, **18**, 1.

KIDD, K.K., KRUGER, S.D., GERHARD, D.S., KIDD, J.R., HOUSMAN, D. & GERTNER, J.M. (1984). Linkage data excluding a locus for Multiple Endocrine Neoplasia Type 2 syndromes from the distal part of the short arm of chromosome 11. *Henry Ford Hosp. Med. J.*, **32**, 262.

KITTUR, S.D., HÖPPENER, J.W.M., ANTONARAKIS, S.E. & 7 others (1985). Linkage map of the short arm of human chromosome 11: location of the genes for catalase, calcitonin, and insulin-like growth factor II. *Proc. Natl Acad. Sci. USA*, **82**, 5064.

KNUDSON, A.G. JR. (1973). Mutation in human cancer. *Adv. Cancer Res.*, **17**, 317.

KOUFOS, A., HANSEN, M.F., LAMPKIN, B.C. & 4 others (1984). Loss of alleles at loci on chromosome 11 during genesis of Wilms' tumour. *Nature*, **309**, 170.

KOUFOS, A., HANSEN, M.F., COPELAND, N.G., JENKINS, N.A., LAMPKIN, B.C. & CAVENEE, W.K. (1985). Loss of heterozygosity in three embryonal tumours suggests a common pathogenetic mechanism. *Nature*, **316**, 330.

KRONTIRIS, T.G., DIMARTINO, N.A., COLB, M. & PARKINSON, D.R. (1985). Unique allelic restriction fragments of the human Ha-*ras* locus in leukocyte and tumour DNAs of cancer patients. *Nature*, **313**, 369.

ORKIN, S.H., GOLDMAN, D.S. & SALLAN, S.E. (1984). Development of homozygosity for chromosome 11p markers in Wilms' tumour. *Nature*, **309**, 172.

PRZEPIORKA, D., BAYLIN, S.B., McBRIDE, O.W., TESTA, J.R., DE BUSTROS, A. & NELKIN, B.D. (1984). The human calcitonin gene is located on the short arm of chromosome 11. *Biochem. Biophys. Res. Commun.*, **120**, 493.

REEVE, A.E., HOUSIAUX, P.J., GARDNER, R.J.M., CHEWINGS, W.E., GRINDLEY, R.M. & MILLOW, L.J. (1984). Loss of a Harvey *ras* allele in sporadic Wilms' tumour. *Nature*, **309**, 174.

RIGBY, P.W.J., DIECKMANN, M., RHODES, C. & BERG, P. (1977). Labelling deoxyribonucleic acid to high specific activity *in vitro* by nick-translation with DNA polymerase I. *J. Mol. Biol.*, **113**, 237.

ROGLER, C.E., SHERMAN, M., SU, C.Y. & 5 others (1985). Deletion in chromosome 11p associated with a hepatitis B integration site in hepatocellular carcinoma. *Science*, **230**, 319.

SCHIMKE, R.N. (1984). Genetic aspects of Multiple Endocrine Neoplasia. *Ann. Rev. Med.*, **35**, 25.

SHIH, C. & WEINBERG, R.A. (1982). Isolation of a transforming sequence from a human bladder carcinoma cell-line. *Cell*, **29**, 161.

SOLOMON, E. (1984). Recessive mutation in aetiology of Wilms' tumour. *Nature*, **309**, 111.

SOUTHERN, E.M. (1975). Detection of specific sequences among DNA fragments separated by gel electrophoresis. *J. Mol. Biol.*, **98**, 503.

VAN HEYNINGEN, V., BOYD, P.A., SEAWRIGHT, A. & 8 others (1985). Molecular analysis of chromosome 11 deletions in aniridia – Wilms' tumour syndrome. *Proc. Natl Acad. Sci. USA*, **82**, 8592.

VASICEK, T.J., McDEVITT, B.E., FREEMAN, M.W. & 5 others (1983). Nucleotide sequence of the human parathyroid hormone gene. *Proc. Natl Acad. Sci. USA*, **80**, 2127.

WILSON, J.T., WILSON, L.B., DERIEL, J.K. & 4 others (1978). Insertion of synthetic copies of human globin genes into bacterial plasmids. *Nucl. Acids Res.*, **5**, 563.